# Measuring the temporal dynamics of inter-personal neural entrainment in continuous child-adult EEG hyperscanning data

**DOI:** 10.1016/j.dcn.2022.101093

**Published:** 2022-02-25

**Authors:** I. Marriott Haresign, E.A.M. Phillips, M. Whitehorn, L. Goupil, V. Noreika, V. Leong, S.V. Wass

**Affiliations:** a University of East London, London, UK; b Department of Biological and Experimental Psychology, School of Biological and Behavioural Sciences, Queen Mary University of London, London, UK; c Department of Experimental Psychology, University of Cambridge, Cambridge, UK; d School of Social Sciences, Nanyang Technological University, Singapore

**Keywords:** Entrainment, Hyperscanning, EEG, Social interaction, Granger causality, Phase Locking Value, Phase Transfer Entropy, Cross-correlations

## Abstract

Current approaches to analysing EEG hyperscanning data in the developmental literature typically consider interpersonal entrainment between interacting physiological systems as a time-invariant property. This approach obscures crucial information about how entrainment between interacting systems is established and maintained over time. Here, we describe methods, and present computational algorithms, that will allow researchers to address this gap in the literature. We focus on how two different approaches to measuring entrainment, namely concurrent (e.g., power correlations, phase locking) and sequential (e.g., Granger causality) measures, can be applied to three aspects of the brain signal: amplitude, power, and phase. We guide the reader through worked examples using simulated data on how to leverage these methods to measure changes in interbrain entrainment. For each, we aim to provide a detailed explanation of the interpretation and application of these analyses when studying neural entrainment during early social interactions.

## 1 Introduction

Behavioural evidence suggests that social factors influence how infants pay attention (Yu and Smith, 2016) and learn (Kuhl et al., 2003) during early life. But we currently understand little about how these interpersonal influences are instantiated in the brain (Wass et al., 2020, Redcay and Schilbach, 2019, Redcay and Warnell, 2018, Hoehl et al., 2021). Hyperscanning is a method of simultaneously acquiring neural activity from two or more individuals that allows insights into these questions (Dumas et al., 2010, Schilbach et al., 2013). Hyperscanning approaches are often paralleled with an emphasis on using more free-flowing 'naturalistic' study designs that record brain activity during real-life interactions – rather than studying neural responses to repetitive and unecological, trial-based tasks administered via a computer.

Recently, research with non-human animals (e.g., Kingsbury et al., 2019; Zhang and Yartsev, 2019) and human adults (Liu et al., 2018, Redcay and Schilbach, 2019), as well as research with children/infants using fNIRS (Nguyen et al., 2021, Piazza et al., 2021, Reindl et al., 2018) and electroencephalography (EEG; Leong et al., 2017; Wass et al., 2018) has started to use hyperscanning to uncover complex patterns of interbrain entrainment (IBE) during social interaction. Research relying on EEG with child/infant populations has shown that bidirectional Granger-causal influences between infants’ and adults’ neural activity are greater in theta (3–6 Hz) and alpha frequency bands (6–9 Hz) during moments of mutual than non-mutual/ averted gaze (Leong et al., 2017). We also know that patterns of IBE in the theta and alpha bands are higher when adults model positive emotions during social interaction than when adults model negative emotions (Santamaria et al., 2020). These findings suggest that - consistent with fNIRS studies that have shown IBE patterns over longer temporal scales (e.g., Piazza et al., 2021; Nguyen et al., 2020, Nguyen et al., 2021) - IBE may also be discernible at the more fine-grained, sub-second scale studied using EEG.

All these approaches used thus far, however, share one fundamental limitation. Hyper-scanning researchers typically calculate the amount of IBE observed between two interacting partners averaged across whole experimental conditions (Pérez et al., 2017, Leong et al., 2017) and even whole interactions (e.g., Kinreich et al., 2017). They then compare IBE values between different conditions, or correlate IBE estimates with an outcome variable (e.g., learning) (Leong et al., 2019). For example, Leong et al. (2017) compared the amount of observed entrainment across all moments of direct vs averted gaze during 5-min social interactions: they collapsed all of their data down to a single IBE value per signal frequency band. A similar approach was taken by Pérez et al. (2017), who compared IBE values estimated separately for different frequency bands and topographical locations, but again without consideration of how IBE varied over time, and how it may have developed over the course of the interaction.

Effectively, therefore, these approaches produce an index of IBE that includes information on how entrainment varies by frequency (e.g. Leong et al., 2017) and by scalp topography (e.g. Santamaria et al., 2020) – but which excludes information on how IBE fluctuates over time. This omission, we argue, fundamentally hinders our understanding of how real-life infant-adult social interactions are substantiated in the brain. The same observation largely holds for most of the hyperscanning research in adult populations, where similar points have been raised concerning the limitations of current approaches (e.g., Novembre and Iannetti, 2020; Moreau and Dumas, 2021).

### 1.1 The importance of the (missing) temporal dimension

Studies using event-related potentials (ERPs) have shown that even young infants’ brains show millisecond-level sensitivity to ostensive signals (e.g., Farroni et al., 2002; Hoehl and Striano, 2008, Hoehl and Striano, 2010, Quadrelli et al., 2019). But this research is all unidirectional: it examines how the recipient of an ostensive signal is influenced by the ‘sender’ of the signal. Very little research has examined the fine-grained temporal dynamics of early social interaction from a bidirectional perspective: by examining how ostensive cues affect the inter-relationship between both partners’ brain activity (Wass et al., 2020).

For example, one early study found that, in the 3–9 Hz range, neural activity in one partner consistently predicted the other partner’s neural activity more strongly during direct compared with indirect gaze (Leong et al., 2017). But how is it mechanistically possible for two brains to influence each other over such fine-grained temporal scales? To answer this question, it would be useful to know how IBE varies over time *within* periods of direct gaze. This would improve our understanding of how, mechanistically, IBE is established and maintained:

I. First, it is possible that, during social interactions, certain shared behavioural events such as the onsets of periods of direct gaze could drive changes in IBE (see e.g., section 2.2.1, 2.2.2). Here, changes in IBE would result from transient intra brain changes in spectral power (e.g., Grossmann et al., 2007) and/or phase (e.g., Rousselet et al., 2007) in both the ‘sender’ and the ‘receiver’ of the social cue. For example, this mechanism could be similar to what has been documented for neural entrainment to speech (e.g., Doelling et al., 2014), whereby the onset of the stimulus/ behavioural event drives phasic changes in the brain and leading to increases in entrainment. Beyond speech or vocalisations, mutual gaze onsets, or touch could also act as salient “edges” that create responses in multiple brains at the same time, leading to event-related increases in IBE. According to this model, IBE would be strongly event-locked, peaking immediately after the onset of the behavioural event and decreasing thereafter. The extent of event-locked changes in IBE around behavioural events might also be mediated by other factors including attention (e.g., Golumbic et al., 2013), comprehension (e.g., Pérez et al., 2019), and environmental factors such partner familiarity (e.g., Reindl et al., 2021, in press).
II. Second, it is possible that turn-taking during social interactions could drive changes in IBE. Here, response preparation or anticipation (e.g., Hamilton, 2021; Hirsch et al., 2017; Kirkland, 2020) and/or mutual sensorimotor predictions (Dikker et al., 2014, Shamay-Tsoory et al., 2019, Hamilton, 2021) could lead to concurrent transient changes in either power or phase in both partners (e.g., Mandel et al., 2016; Bögels, 2020), causing changes in IBE that might peak around these ‘handover’ moments and decrease before and after (e.g., Fig. 2d). This mechanism might also be influenced by factors such as the amount (e.g., Nguyen et al., 2021) and (perceived) quality (e.g., Bloom, 1988) of turn-taking.
III. Third, it is possible that continuous, deterministic intra brain changes, that are not locked to discrete behavioural events but depend on dynamic, gradual changes in the shared environment, lead to gradual, continuous changes in IBE*.* This mechanism might be driven by intra brain responses to shared cognition and/or mental representations. For example, Simony et al. (2016) showed that IBE was increased when participants had a shared understanding of a story (Simony et al., 2016). This mechanism it might also take the form of direct ‘neural mimicry’. For example, Kingsbury et al. (2019) used in vivo electrophysiological recordings to show populations of cells in the dorsomedial PFC that show similar activity when performing an action as when watching it be performed by someone else (Kingsbury et al., 2019). Again, these changes might take the form of changes in power: increases in spectral power throughout an event (e.g., a look/ episode of attention) can increase signal-to-noise ratios and cause changes in sequential IBE (as we show in Section 3.2.1). This is conceivable as, for example, infant theta power increases through an attentional episode (e.g., Jones et al., 2020). Alternatively, gradual changes in frequency, such as the adjustment of the peak frequency of neural oscillations, could lead to increases in concurrent IBE (Section 3.2.2). This is conceivable as, for example, peak alpha frequency can be modulated by task demands (e.g., Samaha and Postle, 2015; Wutz et al., 2018), and recent accounts have theorised that cross-spectrum frequency adjustment at stimulus onset might be a mechanism behind how ERPs are generated (e.g., Burgess, 2012). According to this model, entrainment would increase gradually during a social episode.

Differentiating between these and other hypotheses is essential to understanding how IBE is achieved and maintained. The aim of this paper is to present algorithms that will allow researchers to address this. In Section 1.2 we present an overview of key differences between child and adult EEG that are relevant when conducting hyperscanning developmental research. In Section 2, we present several measures for estimating concurrent (2.2.1, 2.2.2) and sequential (2.2.3, 2.2.4) IBE. Then, in Section 3, we illustrate the ability of each metric to capture IBE using simulated data.

### 1.2 Key differences between child and adult EEG

Researchers analysing EEG recorded from infants and children, and EEG recorded using naturalistic paradigms, face several additional challenges as compared to adult EEG researchers that use screen-based paradigms (Noreika et al., 2020).

Firstly, due to increased movements during the recording. This is challenging because signals generated from movements, such as smiling, vocalisations, eye movements, as well as from the neural processing of each of these behaviours, will contribute to the scalp EEG in a complex way (Georgieva et al., 2020). Although issues of source separation are not new to EEG research, it is known that ICA alone fails to separate different sources in data containing high amounts of movement-related activity (e.g., Plöchl et al., 2012; Dimigen, 2020). This effect is heightened with infant ICA decompositions, for which it is typically harder to identify which components contain predominately artifactual signals and which contain predominately neural signals, compared to ICA decompositions from adult EEG data (Marriott Haresign et al., 2021 in press). For example, even simple artifacts such as blink artifacts can be more clearly differentiated from the ongoing EEG in adult data, allegedly because these movements are more stereotypical and/or produce artefacts of relatively higher amplitude in adults than in infants (Marriott Haresign et al., 2021 in press). This is even more problematic for naturalistic data, during which eye movements would necessarily be less stereotypical than those produced by participants during screen-based tasks.

Secondly, in a traditional, experimenter-designed paradigm, neural responses are examined relative to experimental events. Although evidence suggests that artifacts in traditional experimenter-designed paradigms are still present, and systematically related to experimental events (Yuval-Greenberg et al., 2008), the fact that the experiment (and so the artifacts) follows a consistent structure means that artifacts are relatively easier to deal with. But in a naturalistic paradigm, the events (e.g., eye gaze onsets) are often less systematically related to the artifacts in the data, as there is no clear and consistent temporal structure between spontaneous events and specific artifacts. The future study of IBE using naturalistic paradigms will need to control for the contributions of non-neural signals in the EEG. It may also treat these non-neural signals as data sources, by looking at entrainment between these movement-related signals, e.g., entrainment between EMG associated with facial affect and vocalisations.

An additional challenge is posed by the intrinsic differences in EEG activity that are observed in recordings from children/ infants compared to adults. For example, we know that the speed at which the brain will process information depends on its maturation (e.g., Taylor et al., 2004) and that the canonical frequency bands in child/infant EEG are typically slower than that of adult EEG. For example, peaks in the power density spectrum associated with alpha activity typically observed in the 8–12 Hz range in adults can be seen clearly in one-year-old infant EEG between 6 and 9 Hz and are lower still in younger infants (Marshall et al., 2002). This presents a unique problem for developmental researchers interested in phase entrainment between infant and adult EEG. One solution to this problem might be to use cross-frequency entrainment methods (Noreika et al., 2020) for example cross-frequency phase coupling (see section 2.2.2.2 for further discussion). It is also known that the amplitude of slower oscillations is larger in infant EEG than in adults’, which could arguably affect amplitude-amplitude or amplitude-phase coupling.

## 2 Methods for identifying different types of entrainment between infant and adult EEG data

In this section we present an overview of the different ways of measuring IBE (Section 2.1) and describe how they can be applied to different aspects of the brain signal (amplitude/ power and phase) (Section 2.2). In Section 3 (applied methods) we will guide the reader through application of these methods, using simulated EEG hyperscanning data.

### 2.1 Overview of different ways of measuring inter-brain entrainment (IBE)

**Fig. 1:**
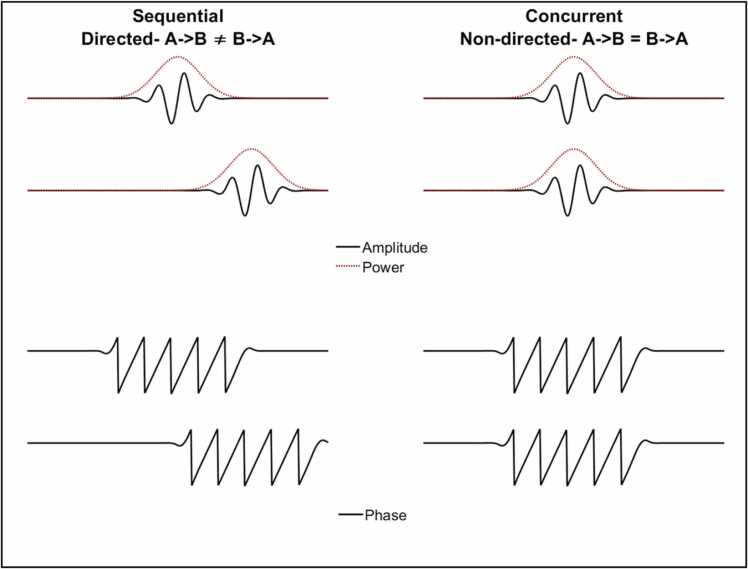
Schematic illustration of the two-entrainment metrics, concurrent and sequential, that we consider in the paper, along with the three aspects of the brain signal: amplitude, power and phase.

Although the term ‘entrainment’ is sometimes used only to describe only *sequential* relationships between two signals, here we use it in a broader sense, to describe any temporally coordinated relationship between two signals. Inter-brain entrainment (IBE) can, then, be measured in two ways. First, concurrent IBE (see Fig. 1) indexes a zero-lag, simultaneous relationship: ‘at times when A is high, B is also high’ or (for a negative relationship): ‘at times when A is high, B is low’. Concurrent IBE is often referred to using the term ‘synchrony’, and is undirected: A->B is indistinguishable from B->A. Second, sequential IBE indexes a lagged, or temporally oriented relationship: ‘changes in A forward-predict changes in B′. Sequential IBE is directed, and as such, unlike concurrent coupling, it can be asymmetrical: it can be true that A forward predicts B without it being true that B forwards-predicts A, and vice versa.

IBE can also be measured across both the temporal and the frequency domain, and thus across multiple aspects of the brain signal: amplitude, power, and phase (see Fig. 1). Power is proportional equal to the amplitude squared and so the two measures are closely related. However, some of the measures we describe (e.g., power correlations, see section 2.2.1) can be applied equally to amplitude and/or power, whereas others (e.g., time domain granger causality, see section 2.2.3) are applied on amplitude and *not* power and therefore we feel that it is important to distinguish between the two.

Currently, most fNIRS and fMRI hyperscanning studies measure co-fluctuations in the amplitude of the signal – which depending on the method, measures blood oxygenation/deoxygenation (fNIRS), the BOLD signal (fMRI) or voltage (for EEG). Currently, most EEG hyperscanning studies examine phase IBE. There appears, however, to be no reason why these trends should not change in the future.

#### 2.1.1 Measuring concurrent IBE of amplitude and power: correlations

Examining concurrent IBE through correlations is one of the simplest and most flexible techniques. Zero-lag concurrent IBE can simply be measured by calculating correlation coefficients between two time series. Spearman’s correlation is generally favoured due to its invariance to non-normally distributed and outlier prone data (Cohen, 2014). The same analysis can be applied either to the amplitude of the brain signal or to the power in particular frequency bands. The accompanying code for this section allows the reader to compute single-trial correlations (Spearman’s rho) at each time-frequency point, between pairs of electrodes (e.g., using data from Cz from person one and Cz from person two).

#### 2.1.2 Measuring concurrent IBE of phase: ITC and Phase Locking Value (PLV)

Phase locking can be estimated in three ways to detect transient phase changes/shifts (often event-locked), or stable phase-coupling across time. First, point-wise phase consistency (e.g., inter trial coherence, ITC) across repeated events can be estimated over time and electrodes within a single brain (see SM section 8). This produces one estimate of phase consistency per time-point which represents the phase distribution across trials for that time-point and is suitable for detecting transient or discrete event-locked phase changes.

Second point-wise phase locking (e.g., Lachaux et al., 1999) can be estimated according to:

(1) $$\mathrm{PL}V_{n}=\left(\frac{1}{N}\right|\sum_{k=1}^{N}{\mathrm{e}}^{i(\phi \left(t,k\right)-\psi \left(t,k\right))}|,$$

Where N is the number of trials, $\phi \left(t,k\right)\;$is the phase on trial *k*, at time *t*, in channel ϕ and ψ(t,k) at channel ψ. This produces one estimate of phase consistency per time-point which represents the phase locking across trials for that time-point and is suitable for detecting transient or highly event-locked changes phase locking. $\mathrm{PL}V_{n}$ varies between 0 and 1 where 1 indicates perfect phase locking over trials and 0 indicates no phase locking over trials.

Third, phase locking can be also measured within a single trial over a defined temporal window (e.g., Tass et al., 1998). This is useful for estimating whether two oscillations are stably phase-coupling to each other during that window. Phase locking withing a temporal window can be measured according to:

(2) $$\mathrm{PL}V_{t}=\left(\frac{1}{T}\right|\sum_{n=1}^{T}{\mathrm{e}}^{i(\phi \left(t,n\right)-\psi \left(t,n\right))}|,$$

Where T is the number of observations or time samples within the window, $\phi \left(t,n\right)\;$is the phase on observation *n*, at time *t*, in channel ϕ and ψ(t,n) at channel ψ. $\mathrm{PL}V_{t}$ varies between 0 and 1, where 1 indicates perfect phase locking over time and 0 indicates no phase locking over time. Both measures can either be computed between a single brain and an external stimulus with a pseudo-periodic structure (e.g., speech), or estimated at the interpersonal level, between two or more brains. Here the focus is on the latter, though mathematically they are identical. The accompanying code allows for analysis of phase locking within (sliding window) and across trials (for each sample). This will allow researchers to look at how changes in IBE fluctuate over time.

It is worth noting any two signals with a common dominant frequency (e.g., two brains with high power in the alpha band) will show relatively consistent variation in phase over time – and hence high phase locking between the signals. This has been used to argue that PLV is particularly prone to detecting spurious hyper connections (Burgess, 2013). However, this is only a problem in a few scenarios. Real EEG data - even after narrow-band filtering - will still have random variations in the phase of the signal over time such that PLV between narrowband filtered signals will also change over time. So, two alpha oscillators will show consistently high PLV only where there is little to no random variations in the phase of both signals over time, which is not a very reasonable assumption for real EEG data. In addition, this problem can be at least partially circumvented by relying on permutation techniques. For instance, PLV between two brains can be measured over time-scrambled and real data: if the phase locking is purely attributable to the fact that both brains oscillate at the same rhythm in a rather constant way, the time-scrambled data and the real data will show similar levels of phase-locking. On the contrary, if a substantial part of the phase-locking depends on the real-time interaction between the two partners, real data will show higher phase locking than scrambled data.

##### 2.1.2.1 Side note on power and PLV: induced versus evoked responses

When analysing any event locked changes in EEG power and/or phase-based entrainment it is important to consider whether these are evoked or induced responses. Evoked responses are additive signals superimposed upon the background/ongoing EEG; induced responses are changes in power and/or phase that happen within the background/ongoing EEG. In other words, evoked responses are transient changes that do not relate to background oscillatory activity, while induced responses entirely depend on the adjustment of background oscillators to incoming stimuli. Whilst changes in power/phase resulting from stimulus-locked **evoked** signals could give the appearance of increased entrainment between two brains, this is interpretationally quite different to potential changes due to **induced** neural activity driving increases in entrainment. For example, if increases in spectral power from two signals are driven purely by evoked and not induced responses then it is incorrect to examine phase resetting as a potential mechanism behind IBE (or phase-locking more generally) and incorrect to use the term neural IBE to refer to these mechanisms (e.g., Keitel et al., 2021).

This problem is further complicated because, as Muthukumaraswamy et al. (2011) show, transient increases in power can lower error in phase estimation and give the appearance of heightened phase locking (see also, Burgess, 2013). Separating increases in power from genuine increases in phase locking is difficult and continually debated (e.g., Sauseng et al., 2007). As a consequence, the best practice for researchers using event-related phase-locking is to always show accompanying power plots and examine power and phase simultaneously.

##### 2.1.2.2 Side note on cross-frequency PLV: dealing with different canonical frequencies

As described above (Section 1.2) the canonical frequency bands in infant EEG are typically slower compared to adult EEG. It may, therefore, be more appropriate for researchers measuring the quantity of phase-locking between infant and adult EEG to use cross-frequency phase locking. Cross frequency phase entrainment or PLV *m:n* is calculated similarly to PLV as follows:

(3) $$\mathrm{PL}V_{\mathrm{mn}}=\left(\frac{1}{N}\right|\sum_{k=1}^{N}{\mathrm{e}}^{i(∆\phi_{k}\left(f_{n},f_{m},t,k\right))}|,$$

Where, *N* is the number of trials and $∆\phi_{k}\left(f_{n},f_{m},t,k\right)$ is calculated as follows:

(4) $${∆\phi }_{k}\left(f_{n},f_{m},t\right)=\;\left(\frac{n+m}{2\cdot m}\cdot \phi \left(f_{m},t,k\right)-\frac{m+n}{2\cdot n}\cdot \psi \left(f_{n},t,k\right)\right),$$

Where *n* and *m* are the centre frequencies of the two signals and should be integer values satisfying the equation $m∙f_{n}=n∙f_{m}$, and $\phi \left(f_{m},t\right)$, is the phase angle at channel ϕ, at time *t,* on trial *k*, and channel ψ. Note that as we have described in section 2.2.2 PLV can be applied over trials (1) or in a time window within a given trial (2). The same applies for cross frequency PLV, although here we only describe the equation for estimating cross frequency PLV over trials (3). Cross frequency phase locking shares the same underlying interpretation as standard phase locking. In accompanying articles in the special edition (Kayhan et al., 2021, in press), we have provided readers with a full pipeline for computing cross-frequency phase-locking within (sliding window) and across trials (for each sample).

#### 2.1.3 Measuring sequential IBE of amplitude and power – Granger Causality (GC)

The simplest way to measure sequential IBE is simply to repeat the Spearman’s correlation described in section 2.2.1 while shifting one time series forwards or backwards in time relative to the other. For example, if we find that the correlation between two time series x and y is stronger when time series x is backward shifted with respect to time series y, compared with when the simultaneous (‘zero-lag’) correlation between the two-time series is examined, then this indicates that changes in x tend to predict changes in y.

Granger Causality is closely related to this approach, but as well as looking at the time-lagged relationship between two time series, it also increases the sensitivity of the prediction by considering how one time-series forwards predict itself over time (known as the autocorrelation). Given two-time series x and y, Granger Causality is a measure of the extent to which time series x can be predicted by previous samples of y above and beyond how well time series x can be predicted by previous samples of x alone.

GC is defined through the log of ratios of error terms between the bivariate and univariate regressive models, following:

(5) $$\mathrm{GC}=1n\left(\frac{\mathrm{var}\left(e_{x}\right)}{\mathrm{var}\left(e_{\mathrm{xy}}\right)}\right),$$

Where $e_{x}$ is the error term obtained from the univariate autoregressive model fit and $e_{\mathrm{xy}}$ is the error term obtained from the bivariate regressive model fit. Again, the same approach can be adopted to look either at the amplitude of the brain signal, or at the power within frequency bands. Time frequency (spectral) GC involves computing the dot product between the regressive coefficients and complex sine waves, (analogous to the Fourier transform) and then applying those results to the error variance via the transfer function (Cohen, 2014).

Finally, Partially Directed Coherence (PDC) is a frequency domain formulation of GC (Sameshima and Baccala, 2014), measured from the coefficients derived from the autoregressive modelling process described above. PDC has also been used to investigate IBE across adult - infant dyads (e.g., Leong et al., 2017; Santamaria et al., 2020). PDC along with other methods of frequency domain entrainment based on autoregressive modelling can be implemented using the extended multivariate autoregressive modelling toolbox (Faes et al., 2013).

##### 2.1.3.1 Side note on EEG data stationarity and GC

Stationarity refers to whether the statistical properties of data change over time. For example, EEG data that contain low-frequency drifts over time can cause data to become nonstationary as the mean of the data is changing over time. Non-stationarity may take various forms. One form of non-stationarity manifest itself through unit root processes - where the data may exhibit a stochastic trend or “random drift”. This form of non-stationarity is common in financial time series (e.g., stock prices), but less common in neuroimaging data (because neurophysiological processes are generally physically constrained). Many stationarity tests (including the KPSS test which is implemented within the MVGC toolbox (Barnett and Seth, 2014)) only test for unit-root stationarity. However, unit-root is not the only kind of non-stationarity; there is also "structurally varying" non-stationarity, which may be more common in ERP or more generally task-related data. Here, the statistical properties of the time series, e.g., mean/standard deviation over time, the amount of periodicity in the data, change over time, either in a structured/deterministic or stochastic way (unit root tests may or may not fail on this form of non-stationarity). This implies that the Granger causality itself may change over time. Overall stationarity in neuroscience for GC is an ongoing problem (e.g., see Barnett et al., 2018a; 2018b). Current common approaches to address non-stationarity in EEG data include polynomial detrending (Seth et al., 2015), and/or subtraction of the averaged ERP from single-trial data (e.g., Wang et al., 2008), but these are limited. Another viable solution to address nonstationary EEG data is to segment the data into shorter time windows in which the data would be stationary enough to perform GC analysis, although this approach needs more empirical testing.

##### 2.1.3.2 Side note on model order and GC

A crucial parameter to consider when using GC analysis is model order. Model order determines the number of previous samples of a time series that will be used in the (bivariate) autoregressive model fit. For instance, if your data is sampled at 1000 Hz, a model order of 5 means that the model will use a weighted sum of the previous 5 ms of data. The model order used will in part determine the frequency precision of the spectral GC estimates. In our example using only 5 ms of data, we would only base our GC estimate on 1/50th of a cycle of 4 Hz activity. To better capture low-frequency dynamics, it can therefore often be useful to downsample the data prior to analysis. For example, if we resampled our data from 512 Hz to 128 Hz, still using a model order of 5 would mean we now consider the previous 39 ms of data in the model fit. Alternatively, one could increase the model order, but models with higher orders typically require longer time segments and more trials as there are more parameters that need to be estimated (Cohen, 2014). Further considering lower frequency dynamics is a good reason to lean towards the highest model order that is appropriate for the data. In our example increasing the model order to 15 would result in us considering the previous 120 ms (at 128 Hz), almost half a cycle of 4 Hz activity. This example illustrates the fact that the model order used can have important consequences on the estimation of GC. Some routines exist to help guide the estimation of model order, the most common being Bayes information criterion (BIC) and Akiake information criterion (AIC). Both are implemented within the MVGC toolbox (Barnett and Seth, 2014).

##### 2.1.3.3 Side note on spectral power and GC

The relationship between spectral power and spectral GC is still uncertain, for example at present it is unclear how changes in spectral power affect GC estimates and anecdotal evidence suggest that it is not uncommon to find correlations between spectral power and (spectral) Granger Causality. Empirical research has shown that increases in event-locked spectral power corresponding to ERPs co-occur with increases in spectral GC (e.g., Wang et al., 2008), but whether these changes in power *caused* the changes in GC or vice versa is uncertain. It will be important for future research to fully explore this relationship (e.g., Winkler et al., 2015). For example, does the strength of the GC scales linearly with the amount of spectral power? And how is this relationship affected by the sampling rate, signal to noise ratios and so on?

#### 2.1.4 Measuring sequential IBE of phase – Phase Transfer Entropy (PTE)

Phase transfer entropy (PTE) allows researchers to measure sequential IBE of phase. It is calculated using the following equation:

(6) $$\mathrm{Phase}\;TE_{x\to y}=H(\theta_{y}\left(t\right),\;\theta_{y}(t^{\prime }))+H(\theta_{y}\left(t\prime \right),\;\theta_{x}(t^{\prime }))-H(\theta_{y}\left(t\prime \right))-H(\theta_{y}\left(t\right),\;\theta_{y}\left(t^{\prime }\right),\;\theta_{x}(t^{\prime })),$$

Where θ(t) is the phase of signal X(t), t′ = t – δ, and $\theta_{x}(t^{\prime })$ and $\theta_{y}(t^{\prime })$ are the previous states of the phase angle time series of x and y, with a given lag of δ. Given two-time series x and y, like GC, transfer entropy (TE) estimates whether including the past of x influences our ability to predict y and vice versa. However, unlike GC, TE does this by comparing conditional probabilities (Lobier et al., 2014). E.g., if a signal X ‘causes’/ ‘disambiguates’ a signal Y, then the probability density of the future of Y conditioned on its past should be different from the probability density of the future of Y conditioned on the pasts of both X and Y. As transfer entropy is based on the same underlying principles as GC, it has been shown that results obtained using GC and PTE are identical for Gaussian variables (e.g., Barnett et al., 2009; Barnett and Bossomaier, 2012). Therefore, results from phase transfer entropy analyses may be interpreted as reflecting directed information flow between two phase angle time series.

Whilst Phase Transfer Entropy has not been widely used within cognitive neuroscience as a framework for analysing entrainment patterns between two systems, it has many advantages and useful properties. For example, as entropy is not based on the temporal structure of the data, it can be computed over time *and* trials, whereas other measures such as PLV can only be computed over time *or* trials, not both. This is a major advantage as including data from time and trials simultaneously means that entropy can be computed in shorter time windows than other window-based entrainment measures (e.g., GC), thus retaining a greater degree of the original temporal precision of the data whilst still having sufficiently high signal to noise ratios (Cohen, 2014).

### 2.2 Cautionary note on the importance of the temporal scale

Many of the metrics described above are highly sensitive to the temporal scale of the analysis. For example, if we observed a transient increase in spectral power in two signals (x and y), where the peak of y occurred a few hundred milliseconds after the peak of x. When using a fine temporal scale (e.g., estimating entrainment in a 200 ms sliding window), we would not detect changes in concurrent IBE, but if we were to use a larger time window (e.g., estimating entrainment in a 1 s sliding window) it is possible to observe changes in concurrent IBE (e.g., see Fig. 6). This we illustrate using simulated data in Section 3.3. This is because, although downsampling can be a useful step in some analysis (e.g., for GC, see section 2.2.3.2), downsampling can create artifacts by spreading the signal in time that can lead to the detection of spurious entrainment.

## 3 Simulations and applied methods for measuring IBE and differentiating event-locked from non-event-locked changes

In this section we present simulations in which we artificially introduced a given relationship between two time series. We then compute the metrics described above to assess how well each metric reflects this relationship. The purpose of this is to guide the reader through application of the various metrics. Throughout the section, we also discuss how different signal changes can manifest either as transient, event-locked changes (Section 3.1) or as more continuous, non-event-locked changes (Section 3.2). In 3.4, 3.5 we describe methods to quantify whether observed event-locked changes in IBE differ significantly from chance.

### 3.1 Simulations - event-locked changes in child-adult neural entrainment

#### 3.1.1 Amplitude and power

At its most simple level, computing the correlation coefficients for time-frequency amplitude/power does not involve any algorithms more complex than a Spearman’s correlation. In the accompanying code, we provide routines for computing concurrent amplitude/power entrainment. However, for the remainder of this section we will focus on GC, which is a more appropriate complex measure for assessing entrainment in EEG data: as detailed above, it not only assesses the potential contribution of one signal to another over time (e.g., the extent to which time series x can be predicted by previous samples of y), but also considers autocorrelations between each signal (e.g., the extent to which x can be predicted by previous samples of x alone).

**Fig. 2:**
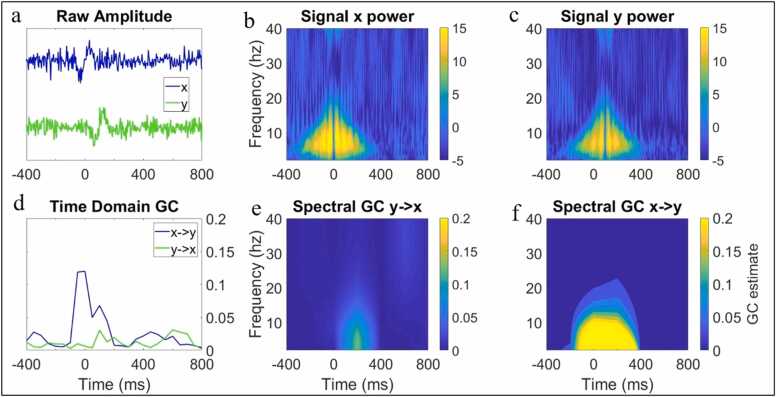
Simulated data showing one mechanism that could give rise to increases in interbrain granger causality between parents and infants. (a) shows the two correlated (single-trial amplitude) transient signals x and y. Y was generated from previous samples of x with a lag of 100 ms, such that (d) there is a substantial event locked increase in GC from x to y but no GC influence from y to x. (b) shows time-frequency power from signal x from panel a. (c) shows time-frequency power from signal y from panel a. (e) shows spectral GC from y to x and (g) from x to y.

To illustrate how event-locked neural responses might give rise to changes in sequential amplitude/power IBE, we simulated two ERP-like signals (x and y) (see Fig. 2). Signal y was generated from previous samples of x plus noise (see SM Section 2 and 7 for full details). From this simulation, it can be seen that the sequential IBE between x and y observable in the raw data (Fig. 2a) manifests as strong x->y GC influences but not y->x GC influences, as expected (Fig. 2d). When the same analysis is applied to the power of the signal (Fig. 2b, 2c), the predicted results are again observed. Spectral x->y GC influences are observed across a range of lower frequencies (Fig. 2e), but no spectral y->x effects are observed (Fig. 2f). In the accompanying code for this section, we provide the user with routines for implementation of time domain and spectral GC (i.e., sequential IBE based on amplitude/power) for measuring event locked changes in EEG hyperscanning data. The user is also able to easily specify more advanced parameters such as the time window size and model order used for the time-varying GC estimates.

#### 3.1.2 Phase

**Fig. 3:**
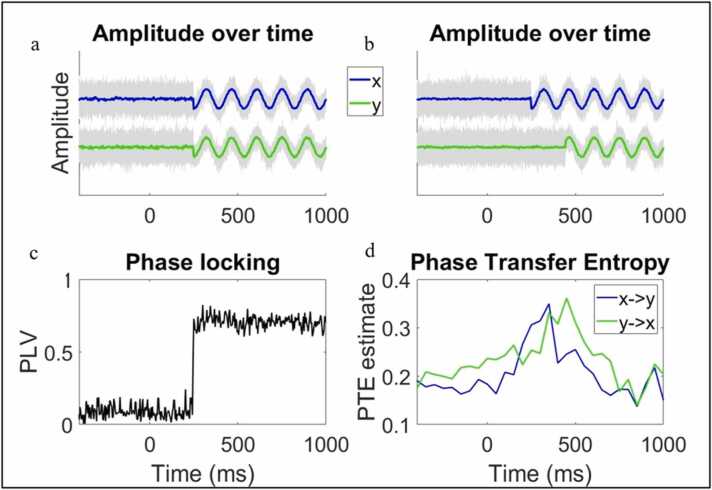
Simulated data showing how event locked phase modulations could give rise to phase-based IBE between two brains. (a) Time series data x and y were subjected to a phase reset at + 200 ms so that they become purely phase locked. Note grey lines show single trial data while blue and green lines indicate data averaged over trials. The increased consistency in phase angle circa + 200 ms between x and y yields (c) a notable increase in the phase-locking between x and y ~200 ms. (b) Simulated data showing a situation in which the phase modulations in one signal (x) predict the phase modulations in another signal (y) and how this lagged/ directed relationship in phase can be captured (d) using phase transfer entropy.

To illustrate how event-locked neural responses might give rise to changes in concurrent phase-based IBE, we simulated 100 trials of two partially phase-locked signals (x and y) with a concurrent phase reset/modulation + 200 ms after an event (time 0) (see Fig. 3a) (see SM Section 4 for more details). From this simulation, it can be seen that, during the time window following the manipulation at + 200 ms, the phase angles of the two time series converge (Fig. 3a) as expected. The phase locking values of the two time series also converge (Fig. 3c) as expected.

To illustrate the sequential phase IBE, we simulated 100 trials of x and y again in the same way, but here the phase modulation in y occurred 200 ms later than in signal x (see Fig. 3b) (see SM Section 5 for more details). From this simulation it can be seen that when phase modulations in one signal occur later/ earlier than phase modulations in another signal (e.g., x in Fig. 3b becomes phase-locked at +200 ms, and y in Fig. 3b becomes phase-locked at +400 ms) and that these modulations are correlated, then this relationship (or form of sequential IBE) can be captured using directed phase IBE methods such as phase transfer entropy – illustrated by the increase in PTE in the time window between phase modulations of x and y (~+200–400 ms) (Fig. 3d). Also note from the simulation that as y becomes phase locked (or entrains to x) at + 400 ms this causes an increase in PTE from y to x. This is because both signals were generated from pure sine waves plus noise and so when y also becomes phase locked at + 400 ms, the activity in y is also now predictive of the activity in x.

In the accompanying code for this section, we provide the user with routines for full implementations of inter-individual, time-frequency PLV and PTE (i.e., sequential IBE based on phase). The user can easily specify parameters such as time window size for PLV/PTE as well as the model order for PTE.

### 3.2 Simulations – non-event-locked changes

#### 3.2.1 Amplitude and power

**Fig. 4:**
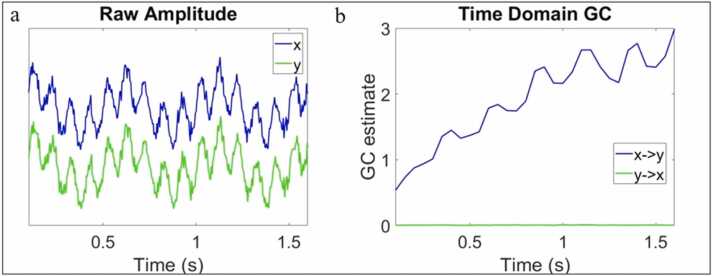
Simulated data showing one mechanism that could give rise to increases in interbrain granger causality between parents and infants. (a) shows two oscillatory signals. Y was generated as a product of previous samples of x with a lag of 25 ms. We decreased the amount of noise in x over time to simulate (b) a gradual increase in GC from x to y throughout the segment.

To illustrate how gradual changes in amplitude/power IBE that are not time-locked to the onset of an event might arise, we simulated two oscillatory signals (x and y) where y was generated from previous samples of x plus white noise (see Fig. 4a). To simulate a gradual change in GC we reduced this noise parameter over time (see SM Section 3 and 7 for more details). From this simulation, it can be seen that, as expected, the x->y GC influence increases during the time window, but no changes in y->x GC influences are observed (Fig. 4b).

In the accompanying code for this section, we show how the same code from the previous Section (3.1.1) can be leveraged to look at questions regarding non-event locked changes in inter brain IBE. No new algorithms are implemented here.

#### 3.2.2 Phase

**Fig. 5:**
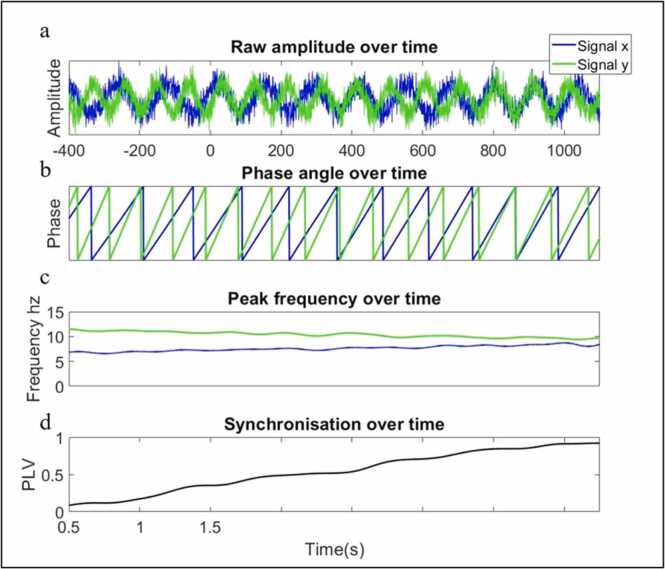
Simulated data showing one mechanism that could give rise to increases interbrain phase locking between parents and infants. (a) Time series data x and y both exhibit slow trends in frequency over time toward a common peak frequency. (c) signal x increases from 6 to 9hz over the time course whereas time series y decreases from 12 to 9hz. The closer the signals become in peak frequency (b) the more consistent the relationship between phase angles over time is, (d) yielding a gradual increase in PLV between x and y over time.

To illustrate how gradual changes in phase IBE might arise that are not time-locked to the onset of an event, we simulated two oscillatory signals (x and y) with slow drifts in peak frequency over time (signal x linearly increased in peak frequency from 6 to 9 Hz and signal y decreased from 12 to 9 Hz) (see Fig. 5). Full details of how we simulated this data, and the time-frequency decomposition can be found in the supplementary materials (SM 6 and 7). From this simulation, it can be seen that the closer the signals become in peak frequency the more consistent the relationship between phase angles over time is, and thus the higher the phase-locking value between x and y is.

In the accompanying code for this section, we show how the same code from the previous Section 3.1.2 can be leveraged to look at questions regarding non-event locked changes in inter brain entrainment. No new algorithms are implemented here.

### 3.3 Simulation- cautionary note on the importance of the temporal scale

**Fig. 6:**
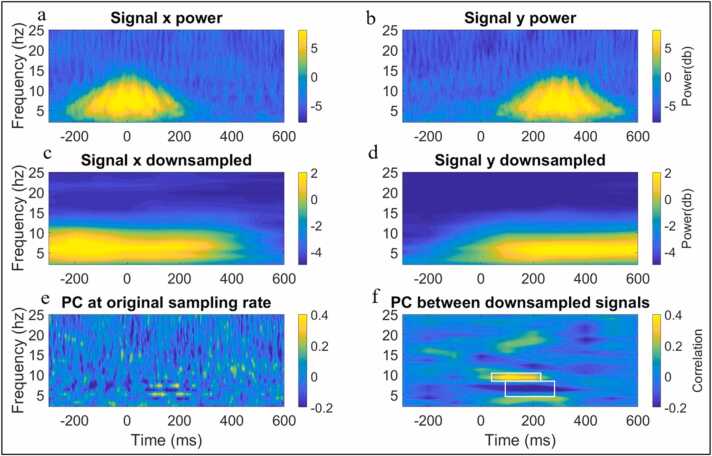
Simulation illustrating the importance of using an appropriate time window for calculating concurrent entrainment. Panel a shows the time-frequency power of signal x. Panel b shows the time-frequency power of signal y. Panel c shows the down sampled (using moving window average) time-frequency power of signal x. Panel d shows the down sampled, time-frequency power of signal x. Panel e shows concurrent IBE (spearman’s correlation of single trial power (PC) between x and y) computed at each time-frequency point (i.e., original temporal scale of data). Panel f shows the same concurrent IBE but computed on the down sampled data. The AOIs on panel f indicate regions of significant correlations.

In this section we highlight how the temporal scale of the analysis influences concurrent IBE estimates. To illustrate this, we simulated two signals that show an event-locked transient increase in spectral power which peaks 300 ms later in signal y compared with signal x (Fig. 6a, 6b). Details of the time-frequency decomposition can be found in SM 7. To compute concurrent IBE, we performed two calculations: first, we calculated Spearman’s correlations between the power of time series x and y at each time-frequency point independently (see Fig. 6e). Second, we down sampled the data using a 0.5 s sliding window with 200 ms of overlap between successive windows (Fig. 6c, 6d) before repeating the same analysis (Fig. 6f). When using a fine temporal scale, we detect no changes in concurrent IBE, but when using a larger time window (reduced temporal precision) we do (Fig. 6f). This illustrates how the pre-processing of data prior to IBE analyses can alter the results.

### 3.4 Quantifying event-locked changes in child adult neural entrainment

In this final section we consider the question of statistical significance. If we know that specific events occurred in the data, how can we test whether statistically significant changes in IBE occurred relative to these events?

#### 3.4.1 Amplitude and power

Significant changes in GC can be evaluated with F-statistics, which is implemented through the MVGC toolbox (Barnett and Seth, 2014). Statistical significance can also be obtained via nonparametric permutation testing, which can be applied to power correlations, time domain and spectral GC (Maris and Oostenveld, 2007). The benefit of the latter measure is that it also deals with the problem of multiple comparisons. For measuring changes in GC that are strongly time/event locked, permuting the order of the time segments within trials is generally recommended over permuting the trial order whilst leaving the time segments intact (Cohen, 2014).

#### 3.4.2 Phase

Assuming a von Mises distribution (normal distribution for circular data) statistical significance of ITC and PLV can be evaluated against a p-value, approximated against the null hypothesis using the Rayleigh’s test which can be implemented using the Circstat toolbox (Berens, 2009). Statistical significance can also be assessed against a threshold ITC/PLV value (Cohen, 2014). Any values which exceed this resulting threshold can be considered significant. Alternatively, the significance of time-frequency varying ITC and PLV as well as PTE (when computed in a sliding window within trials) can also be assessed using nonparametric permutation testing (e.g., Maris and Oostenveld, 2007).

### 3.5 Correcting for multiple comparisons when measuring changes in parent-child neural entrainment

When analysing EEG data, we are typically interested in how a given effect varies as a product of time, frequency, and topography. This makes exploratory EEG analysis susceptible to the problem of multiple comparisons: increasing the number of statistical inferences drawn from the data will also increase the likelihood of obtaining a significant result. There are several approaches to correct for this problem. For example, if you are only testing a limited number of regions/frequencies of interest it is appropriate to use the Bonferroni correction method. For more complex comparisons involving a large number of channels and time-frequency points, Bonferroni correction is not appropriate. In these situations, correction for multiple comparisons should be made using pixel or cluster-based permutation statistics (Maris and Oostenveld, 2007). For a more detailed discussion of which correction methods to use when we refer the author to Cohen (2014), chapter 33).

## 4 Discussion

*Procambarus clarkia*, a breed of freshwater crayfish, exhibit only a small range of social behaviours, primarily focussed on dominance/ subordination, yet their physiological systems are capable of supporting these interactions as well as intra-individual interactions with their environment with a remarkable level of temporal fidelity (e.g., Schapker et al., 2002). The way that humans interact with their environment and each other is infinitely more complex and multi-layered (Hasson and Frith, 2016, Hoehl et al., 2021, Murray et al., 2016). However, most researchers who study interacting humans during social engagement typically do so using methods that measure how entrainment varies by frequency/topography and between different experimental conditions (or participants), but which obscure how entrainment varies over time. In this article, we have argued that this omission prevents us from developing a mechanistic understanding of how neural entrainment is established and maintained.

In this article, we presented algorithms that allow researchers to measure how entrainment between two brains (or more generally physiological systems) varies as a function of time. We have differentiated between two types of entrainment (Section 2.2): concurrent (‘when A is high, B is high’) and sequential entrainment (‘changes in A forward-predict changes in B′). And we have described how these measures can be applied to three aspects of the neural signal: amplitude, power and phase (Section 2.2, see Fig. 1).

We hope that this guided simulation study and tutorial will help facilitate further research into the possible mechanisms underpinning child-adult neural entrainment. For example, measuring changes in concurrent entrainment of amplitude and power using correlations (section 2.2.1), or of phase using PLV (section 2.2.2) might be used to explore the possibility that certain behavioural events during social interactions could lead to local increases in IBE, for example around moments of mutual gaze onsets or vocalisations (see Section 1.1).

Further measuring changes in sequential entrainment of amplitude and power using GC (section 2.2.3), or of phase using PLV (section 2.2.2) or PTE (section 2.2.4), might be used to explore the possibility that response preparation or anticipation and mutual prediction might lead to changes in sequential IBE (see Section 1.1). This could in theory involve concurrent, transient changes in either power or phase in both partners (e.g., Mandel et al., 2016; Bögels, 2020), that would drive changes in sequential IBE.

Lastly, the same methods described above might be used to explore the possibility that continuous intra brain changes, that are not locked to behavioural events, could also lead to gradual changes in IBE. This might be substantiated for example, through shared cognition and/or mental representations or through direct ‘neural mimicry’, even in the absence of explicit turn-taking (Hamilton, 2021, Kingsbury et al., 2019). These concepts are discussed further in Section 1.1. Alternatively, gradual changes in phase, such as the adjustment of the peak frequency of neural oscillations, could lead to increases in concurrent IBE of phase (Section 3.2.2), through, for example, concomitant modulations of peak alpha frequency in response to task demands (e.g., Samaha and Postle, 2015; Wutz et al., 2018).

Overall, the study of child-adult neural entrainment is still in its infancy and many very basic questions regarding how changes in inter brain entrainment are substantiated at the neural level, and are mediated through behaviour, remain unanswered. It is our hope that the material presented in this paper will aid researchers in addressing these fundamental questions.

### 4.1 Outstanding issues

There are, of course, many outstanding issues with the analysis of EEG hyperscanning data. Substantial questions remain about how successfully artifacts can be removed from brain data (Section 2.1); in understanding the relationship between power changes and IBE (sections 2.2.2.1 and 2.2.3.3) and the problems associated with non-stationarity in EEG data and Granger Causal analyses (section 2.2.3.1); and so on.

Two further outstanding issues should be noted. First, we are often considering events that have different periodic structures, and that unfold over different time scales. For example, researchers might want to examine the relationship between eye gaze shifts (which take place every ~300 ms – i.e., at ~3 Hz), changes in autonomic arousal (between ~0.01and ~0.5 Hz) and changes in EEG (between ~2 Hz-~30 Hz). Although we have presented some methods for looking at this – such as temporal correlations in power at different frequencies (section 2.2.1) and cross-frequency PLV (section 2.2.2.2) – we have not discussed other approaches, such as phase-amplitude coupling (Canolty and Knight, 2010, Tort et al., 2010) that would also be useful.

Second, we have concentrated exclusivity on IBE in relation to bivariate behaviours (e.g., mutual gaze). Unlike the ways in which social information processing is typically studied (i.e., using repeated, discrete and unecological screen-based stimulus), real social interactions involve highly layered and complex sequences of multimodal events that can unfold over multiple time scales in a continuous and interdependent way. For example, consider the multimodal pathways to joint attention as illustrated by Yu and Smith (2016), in which sequences of social interactions between parents and infants often involve initiating and responding to various postural and gestural movements, as well as visual (gaze) information and vocalisations, presented in combination. Future work will require more advanced data analysis and the collection of larger datasets, to explore IBE in relation to more complex multivariate behavioural datasets (for example those modelling gaze, touch, affect and vocal data simultaneously).

## 5 Conclusion

The focus on fine-grained neural responses that we advocated in this paper has the potential to provide valuable new insights into the neural processes that support dynamic social interaction, beyond what is possible using the current/ standard approaches adopted in EEG hyperscanning studies. It is our hope that the considerations that we highlighted, and the methods that we described, will pave the way for future studies which will analyse in more depth the rich temporal dynamics of neural activity, bringing us closer to the true complexity of brain functioning.

## Implementation

All MATLAB functions for the implementation of the various algorithms presented here for event locked entrainment analysis of EEG hyperscanning data, including a sample dual EEG dataset, as well as, code for generating the simulated data can be found here [https://github.com/Ira-marriott/Using-dual-EEG-to-analyse-event-locked-changes-in-child-adult-neural-entrainment-.git].

## Declaration of Competing Interest

The authors declare that they have no known competing financial interests or personal relationships that could have appeared to influence the work reported in this paper.

## Data Availability

Non-identifiable sample data (e.g., EEG data) is available from the corresponding author upon request.
